# ABBV-176, a PRLR antibody drug conjugate with a potent DNA-damaging PBD cytotoxin and enhanced activity with PARP inhibition

**DOI:** 10.1186/s12885-021-08403-5

**Published:** 2021-06-09

**Authors:** Mark G. Anderson, Qian Zhang, Luis E. Rodriguez, Claudie M. Hecquet, Cherrie K. Donawho, Peter J. Ansell, Edward B. Reilly

**Affiliations:** 1 AbbVie Inc., Oncology Discovery, 1 North Waukegan Rd., North Chicago, IL 60064–6099 USA; 2 Formerly AbbVie, Oncology Discovery, 1 North Waukegan Rd., North Chicago, IL 60064 USA

**Keywords:** Prolactin receptor *PRLR*, Antibody drug conjugate, Pyrrolobenzodiazepine dimer, Combination therapy, Poly (ADP-ribose) polymerase I

## Abstract

**Background:**

Prolactin receptor (PRLR) is an attractive antibody therapeutic target with expression across a broad population of breast cancers. Antibody efficacy, however, may be limited to subtypes with either PRLR overexpression and/or those where estradiol no longer functions as a mitogen and are, therefore, reliant on PRLR signaling for growth. In contrast a potent PRLR antibody-drug conjugate (ADC) may provide improved therapeutic outcomes extending beyond either PRLR overexpressing or estradiol-insensitive breast cancer populations.

**Methods:**

We derived a novel ADC targeting PRLR, ABBV-176, that delivers a pyrrolobenzodiazepine (PBD) dimer cytotoxin, an emerging class of warheads with enhanced potency and broader anticancer activity than the clinically validated auristatin or maytansine derivatives. This agent was tested in vitro and in vivo cell lines and patient derived xenograft models.

**Results:**

In both in vitro and in vivo assays, ABBV-176 exhibits potent cytotoxicity against multiple cell line and patient-derived xenograft breast tumor models, including triple negative and low PRLR expressing models insensitive to monomethyl auristatin (MMAE) based PRLR ADCs. ABBV-176, which cross links DNA and causes DNA breaks by virtue of its PBD warhead, also demonstrates enhanced anti-tumor activity in several breast cancer models when combined with a poly-ADP ribose polymerase (PARP) inhibitor, a potentiator of DNA damage.

**Conclusions:**

Collectively the efficacy and safety profile of ABBV-176 suggest it may be an effective therapy across a broad range of breast cancers and other cancer types where PRLR is expressed with the potential to combine with other therapeutics including PARP inhibitors.

**Supplementary Information:**

The online version contains supplementary material available at 10.1186/s12885-021-08403-5.

## Background

Worldwide, over 2.1 million women are impacted by breast cancer each year, causing the most cancer-related deaths among women. In the U.S., over 275,000 women are expected to be diagnosed with invasive breast cancer this year, and over 1 in 8 women will develop the disease over her lifetime, making it the second most diagnosed cancer next to skin cancer for women [1, 2]. Although development of targeted agents has led to improved outcomes in patients with breast cancer, 50% of patients with advanced disease treated with current standard of care will suffer from disease progression [3]. This observation highlights the need for additional treatment options for patients with advanced breast cancer including Her2+, TDM-1 refractory breast cancer and triple negative breast cancer (TNBC). Evolving evidence suggests that therapies targeting the prolactin receptor (PRLR) may provide a distinct advantage in treating these patient populations [4–6].

PRLR is a type 1 cytokine receptor implicated in the initiation and progression of breast cancer [7–10]. The PRLR/PRL (prolactin ligand) signaling axis contributes to estrogen-insensitive breast cancer growth and development. PRLR is expressed in many human breast tumors where its levels are elevated compared to normal breast tissue [11, 12]. High circulating levels of its ligand, PRL, correlate with increased risk for developing breast cancer [13] and with disease progression and reduced response to tamoxifen [13–16]. PRLR has been shown to confer resistance against chemotherapeutic agents including docetaxel, doxorubicin and cisplatin [17].

The functional attributes and tumor expression profile of PRLR make it an attractive target for therapeutic intervention in breast cancer and potentially other malignancies. For example, the neutralizing antibody LFA-102 antagonizes PRLR signaling and proliferation in breast cancer cells and regresses PRL-dependent tumor xenografts [10]. As a large percentage of breast tumors express estrogen receptors and remain sensitive to estrogen, an unconjugated PRLR antibody would be anticipated to be effective primarily only in those breast tumor subtypes where estradiol no longer functions as a mitogen and are, therefore, reliant on PRLR signaling for growth. While LFA-102 was well tolerated as an unconjugated antibody in Phase 1 clinical trials in patients with metastatic breast and prostate cancer, efficacy was limited [18].

In contrast, an antibody drug conjugate (ADCs) targeting PRLR may provide superior therapeutic outcomes including and extending beyond estradiol-insensitive breast cancer populations since its mechanism of action is not dependent on hormonal dependency. Elevated expression of PRLR in tumor versus normal breast tissue may enable an acceptable therapeutic window for PRLR ADCs. An anti-PRLR ADC comprised of a high-affinity function blocking anti-PRLR IgG1 antibody (REGN2878) conjugated to the cytotoxic maytansine derived DM1, has shown significant anti-tumor activity against several breast cancer xenograft tumor models although the activity was limited to those models with high levels of PRLR expression [4].

Herein we describe the characteristics and functional attributes of ABBV-176, a novel pyrrolobenzodiazepine (PBD) ADC targeting PRLR across multiple breast cancer models including low PRLR models and models insensitive to MMAE (monomethyl auristatin)-based PRLR ADCs. Ultimately, the full clinical potential of the PRLR ADC may be realized by combinations with other therapeutics that complement the PBD MoA. Consequently, studies combining PRLR-PBD ADCs with other therapeutics including potentiators of DNA damaging agents were also evaluated.

## Methods

### Antibodies and reagents

A panel of PRLR-specific mAbs was generated using standard hybridoma technology following immunization with the extracellular domain (ECD, 25–234) of PRLR. Candidates were screened based on binding properties, and epitope, and candidates were humanized as IgG1 isotypes before conjugation to MMAE [19]. The lead mAb, h16f, was identified based on improved affinity, epitope binding properties and activity, and then engineered to include an S238C point mutation (S239C based on Kabat numbering system [20]] to permit site-specific DAR 2 conjugation to a PBD dimer (SGD-1882) via mc-Val-Ala linker as previously described [21].

Recombinant human, cynomolgus, and murine PRLR extracellular domains with a His-tag (huPRLR_25–234,_ cyPRLR_25–234_ and muPRLR_20–229_) were expressed in and purified from HEK293 cells. Recombinant ECD of rat PRLR (residues 20–229 fused with poly-His tag at C-terminal), was purchased from Sino Biological Inc. and further purified by gel filtration using Superdex200 (GE Healthcare) in 10 mM HEPES, pH 7.4, 150 mM NaCl, 3 mM EDTA.

### Surface plasmon resonance: human and cynomolgus PRLR extracellular domain binding assay

Binding kinetics of ABBV-176 and the h16f parental mAb to recombinant human and cynomolgus PRLR ECD were determined by surface plasmon resonance-based measurements made on Biacore T200 instrument (GE Healthcare) using previously described methods [22].

### Cell culture

Human tumor cell lines from ATCC were expanded in culture upon receipt and cryopreserved to provide cells at a similar stage passage for all subsequent experiments. For cell lines not authenticated in the 6 months before use, their PRLR expression levels were confirmed by FACS analysis. T47D and 22RV1 were maintained in RPMI-1640 (Gibco Invitrogen, #11875) supplemented with 10% fetal bovine serum (FBS). Cell lines CAMA1, AN3CA BT-474, MCF7, MDA-MB-361, SKBR3, UACC812, MDA-MB-231, SMOV2 [23], SW403, HuH-7, HepG2 and HEK-293 were cultured in DMEM (Gibco-Invitrogen, #11965) supplemented with 10% FBS. All cell culture was done at 37 °C in a humidified incubator with 5% CO_2_. All non-transformed cells were cultured using manufacturer-recommended conditions: NHBE, HUVEC, HMEC, PrEC, and NHDF were obtained from LONZA; primary normal HUF, HRE, MCF10A, HMVEC, and THLE-3 cells were obtained from ATCC; and HRMC, were obtained from iXCells Biotechnologies. All assays were performed within two passages after thawing.

### Retroviral infection

Retroviral infections were performed using the Lenti-X viral system (Clontech) according to manufacturer’s instructions, with PRLR sequences from human and cynomolgus monkey cloned into the pLVX-IRES-puro vector.

### Binding ELISA and fluorescence-activated cell sorting (FACS) analysis

ELISA binding assays with PRLR ECD were performed as previously described, except 0.25 μg/ml of ECD protein was bound to plates [24].

For cellular binding studies, cells were harvested and sorted as previously described [24] using anti-PRLR (mouse version of h16f) or control antibody ((−)Co). When necessary to dissociate cell aggregates, cells were briefly treated with Accutase (Millipore, #SCR005). Data was analyzed using Becton Dickinson FACSDIVA software. For quantitative determination of PRLR on the cell surface, QIFIKIT (Dako) was carried out according to the manufacturer’s instructions, as previously described [25].

### Cytotoxicity assay

Cell lines were plated at 500–5000 cells/well for CellTiter-Glo assays as previously described [25], including a huIgG1-PBD as a negative control ADC.

### Western blot comparison for PRLR expression

Two to three replicate untreated tumors were subjected to Western blot analysis as previously described [24] with anti-PRLR (inv359200, Invitrogen) and anti-actin (Sigma, # A5441). To assess relative PRLR expression levels for xenograft lysates, the low PRLR-expressing MCF7 xenograft (~ 8000 receptors/cell in culture) lysates were run in parallel as an internal standard. For each individual MCF7 lysate, the total PRLR signal was divided by the actin signal and the average value then used to normalize the other CDX and PDX lysates relative to the MCF7 PRLR value.

### In vivo studies

Female SCID (Huh-7 LOT, *n* = 9 per dose group and HepG2, *n* = 10 per dose group) and SCID Beige (BT-474, *n* = 10 per dose group) mice were obtained from Charles River Laboratories (Wilmington, MA) and handled and studies carried out as previously described [25] with the following exceptions. For BT-474.FP2 (BT-474 sub-line) and HepG2, 5 × 10^6^ viable cells were inoculated subcutaneously (s.c.), and for HUH-7 LOT (liver orthotopic subline), 1 × 10^6^ for cells were similarly inoculated. Animals were tagged and followed individually throughout the experiment and dose groups were caged together. Randomization by the matched distribution algorithm and tumor volume calculations, two times weekly, used Study Director version 3.1.399 (Studylog Systems, Inc., South San Francisco). Mice were euthanized by isofluorane inhalation with no oxygen mixture to anesthetize the animals until they became unconscious and stopped breathing in compliance with AbbVie’s Institutional Animal Care and Use Committee guidelines, when tumor volume was ≥1500 mm^3^ or skin ulcerations occurred.

The PDX *N* = 3 study (with single animals, in individual HEPA ventilated cages) was conducted using similar methodologies at Champions Oncology (Baltimore, MD) in compliance with Champion’s Institutional Animal Care and Use Committee and the National Institutes of Health Guide for Care and Use of Laboratory Animals guidelines in a facility accredited by the Association for the Assessment and Accreditation of Laboratory Animal Care [26]. Investigators at Champions were blinded to the identity of the dosing materials.

The expanded CTG-0869 (*n* = 10) and CTG-0670 (*n* = 8) studies were conducted similarly with the following modifications. PDX tumors grown in SCID Bg female were harvested when they reached a size of approximately 1000 mm^3^ size and finely chopped into a homogenous brie. PDX brie (0.2 ml of a 1:1 mixture of brie and Matrigel (BD Biosciences)) was inoculated s.c. into the lower right flank of recipient SCID Bg female study animals. Mice were treated intraperitoneal with the indicated doses of ADCs, and in combination studies with 200 mkd of veliparib administered orally b.i.d for 21 days. Veliparib, synthesized at Abbott Laboratories (Abbott Park, IL), was formulated in 0.9% saline.

### Statistical analysis

IC_50_ and EC_50_ values were determined by nonlinear regression analysis of concentration response curves using GraphPad Prism 6.0 (GraphPad Software, La Jolla, CA). For tumor studies, mean tumor volume and standard error shown were analyzed using the Student’s t-test for differences in T/C values (StatView, SAS Institute, Cary, NC). For the PDX *N* = 3 screening study, the selectivity and anti-tumor activity efficacy of ABBV-176 was by the Welch t-test correlating the log transformed relative tumor volume at the time of maximum tumor growth inhibition (TGI), and mixed effect modeling, comparing the log of fold change in tumor volume (versus the first time point) for every available time point using R software (Bell Laboratories, Madison, WI). Both tests were used, with ≥1 significant test describing treatment efficacy. Survival curves were generated and analyzed using the JMP statistical software v7 (SAS institute, Cary, NC). The Log-Rank (Mantle-Cox) test was used to calculate the survival improvement of treatments.

## Results

### Generation of a PRLR ADC

Candidate antibodies were initially conjugated to MMAE payloads and evaluated for their ability to inhibit the growth of the BT-474, an ER+, progesterone receptor (PR)-, HER2+ breast cancer cell line. BT-474 has ~ 10,000 PRLR receptors per cell, which is lower than the number typically necessary to mediate effective ADC killing, suggesting that efficient internalization may be a critical component for activity of a PRLR ADC (Table 1). This tumor cell line may, therefore, also serve as a surrogate measure of ADC internalization properties. Based on cytotoxic assay results with BT-474, the h16f maleimide-conjugated mc-Val-Cit-MMAE DAR4 ADC was identified as the lead candidate with the most potent inhibitory activity. An antibody with an engineered cysteine (C239) was generated to permit site-specific malemide conjugation of the PBD dimer with DAR 2 and the final ADC conjugate was designated as ABBV-176.

**Table 1:** PRLR Expression and ABBV-176 Cytotoxicity in Human Tumor Cell Lines

	PRLR Expression^**a**^	ABBV-176 IC_**50**_ (nM)^**b**^	h16f-MMAE IC_**50**_ (nM) ^**b**^
Breast cancer			
T47D	26,000	0.0055 (0.006)	0.22
CAMA1	10,000	0.01	5.2
BT-474	10,000	0.24 (0.04)	0.56
MCF7	8000	0.32 (0.3)	> 22
MDA-MB-361	5–10,000	0.77 (0.2)	0.96
SKBR3	5–10,000	0.26	3.67
UACC812	~ 3500	> 22	> 22
MDA-MB-231	Below detection	> 22	> 22
Prostate			
22RV1	8000	0.01	> 22
Endometrial			
AN3CA	8300	0.6	22
Ovarian			
SMOV2	~ 2300	0.16	> 22
Colorectal			
SW403	11,000	0.11	17
Liver			
HepG2	n.d.	8.6	> 22
HuH-7	~ 14,000	5.2	> 22

*IC*_*50*_ half maximal inhibitory concentration, PRLR prolactin receptor, *n.d.* not done due to cell aggregation

^a^Cell surface PRLR per cell is indicated based on quantitative FACS

^b^Cell viability was determined following incubation with indicated ADC for 144 h. The values represent IC_50_s. Averages are shown when multiple experiments were performed, with standard deviations in parentheses. Unconjugated anti-PRLR antibody does not inhibit growth of any of these cell lines

### Binding properties of ABBV-176 for PRLR

**Fig. 1:**
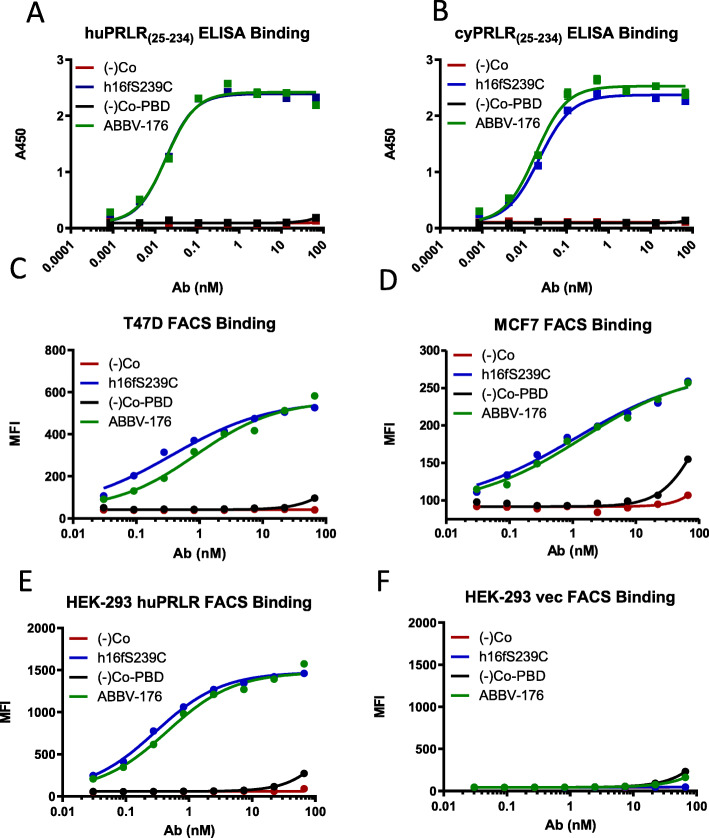
ABBV-176 Binding to PRLR. Binding to immobilized PRLR extracellular domain recombinant protein by ELISA is shown in for ABBV-176 and its unconjugated antibody, along with control antibody (unconjugated and PBD-conjugated) to human (**A**), cynomolgus (**B**) proteins ECDs. Binding to cells expressing PRLR was assessed by fluorescence activated cell sorting (FACS) analysis of the PRLR-high cell line T47D (**C**) and the PRLR low cell line MCF-7 (**D**) with titration curves for geomeans plots shown. Specificity of binding of the anti-PRLR antibody and ABBV-176 is shown in HEK-293 cells engineered to over express PRLR (**E**) and not in negative control HEK-293 cells (**F**)

The binding of both parental antibody h16f and ABBV-176 were similar to both human and cynomolgus monkey recombinant PRLR extracellular domain (25–234), with high apparent affinity (EC_50_ approximately 20 and 18 pM, Fig. 1A and B) and the absence of appreciable binding to mouse or rat PRLR ECD (EC_50_ > 67 nM) by ELISA. As measured by Biacore analysis, the affinities of h16f and ABBV-176 to the recombinant form of the human PRLR ECD were comparable (KD of 1.0 nM), and similar to cynomolgus PRLR ECD (0.7 nM).

Binding of h16f and ABBV-176 to cell surface PRLR was measured by fluorescence activated cell sorting (FACS). Both h16f and ABBV-176 bind comparably to cells expressing human wild-type PRLR including a high expressing tumor cell line (T47D Fig. 1C), a low expressing tumor cell line (MCF-7, Fig. 1D) and HEK-293 cells engineered to express human PRLR (Fig. 1E). No significant cell surface binding was observed with the control antibody or its PBD conjugate (Fig. 1C-E), and neither h16f nor ABBV-176 appreciably bound to control HEK-293 vector control cells (Fig. 1F).

### In vitro potency of ABBV-176 against tumor cell lines and correlation with PRLR expression

h16f-MMAE and ABBV-176 conjugates were evaluated for their ability to inhibit the growth of a panel of 25 breast cancer cell lines expressing different levels of PRLR (Table 1). Measurement of PRLR receptor densities on these tumor cells permitted a preliminary assessment of the correlation between receptor expression and sensitivity to ADC-mediated killing. Results indicated cell line sensitivity to killing by the ADC correlated with PRLR mRNA and protein expression. PRLR was overexpressed in both HER2+ and HER2- tumors. Every tumor cell line sensitive to killing by h16f-MMAE ADC was equally or more sensitive to killing by ABBV-176. Additionally, several tumor cell lines with lower levels of PRLR were sensitive to killing by the PBD conjugate but were largely insensitive to h16f-MMAE. These results suggest that ABBV-176 (h16f (S239C)-PBD) with a payload more potent than h16f-MMAE, has the potential to expand the breadth of sensitive tumors.

A panel of normal cell lines representing kidney, breast, liver, lung, prostate, and vascular endothelium, (HUVEC, HUF, HRE, HMEC, NHBE, PrEC, HMVEC, THLE-3 and MCF-10A) was tested for presence of PRLR, and minimal PRLR protein was observed by Western blot. Consistent with this result, these cells were largely unaffected by treatment with ABBV-176, similar to the non-targeting control ADC, and had much less cell killing than free PBD cytotoxin (Supplemental data).

Since the PRLR/PRL axis has been implicated in other tissues, including reproductive tissues and prostate [9, 27–31], h16f-MMAE and ABBV-176 conjugates were evaluated for their ability to inhibit the growth of non-breast-derived tumor cell lines (Table 1). Although the levels of PRLR RNA in these cell lines was generally much lower than observed for many breast cancer cell lines, several cell lines including ovarian, endometrial, prostate and colorectal cell lines were sensitive to killing by ABBV-176, but not h16f-MMAE. These results suggest that the activity of ABBV-176 may extend to other PRLR-expressing tumor indications beyond breast cancer.

### ABV-176 in vivo efficacy in PRLR-expressing tumor models

**Fig. 2:**
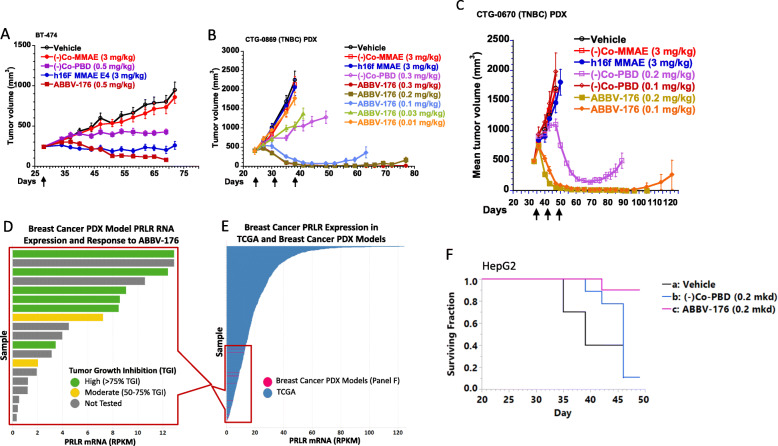
ABBV-176 efficacy against human tumor xenograft models in vivo. The in vivo tumor growth inhibition is shown for tumors in mice dosed as indicated by the arrows with the specific therapeutic or IgG matched non-targeted controls ((−)Co ADCs) as indicated in the legends of each panel. Tumor volumes are shown as mean ± S.E.M. (**A**) Mice implanted with BT-474 breast cancer model and dosed with a single dose of ABBV-176 at 0.5 mg/kg, h16f-MMAE ADC at 3 mg/kg, or matching controls (groups of *n* = 10). (**B**) BR-0869 TNB PDX tumor-bearing mice were dosed Q7D × 3 with ABBV-176 between 0.3 mg/kg and 0.01 mg/kg or 3.0 mg/kg of h16f-MMAE ADC (groups of *n* = 10). (**C**) Mice implanted with breast PDX tumor CTG-0670 were dosed with ABBV-176 or control PBD ADC at 0.2 mg/kg or 0.1 mg/kg, and h16f-MMAE at 3 mg/kg, all at Q7D × 3 (groups of *n* = 8). PRLR expression levels for 8 of the PDX models tested are shown in (**D**) and those expression values are shown relative to the TCGA breast cancer data for PRLR in (**E**). (**F**) Survival curves are shown for mice implanted with the HepG2 HCC model (groups of *n* = 10), where mice were dosed Q7D × 3 with vehicle, 0.2 mg/kg control PBD, or 0.2 mg/kg ABBV-176

The in vivo efficacy of ABBV-176 was evaluated in the BT-474 FP2 human xenograft breast tumor model. BT-474 is a low PRLR-expressing tumor cell line with ~ 10,000 receptors per cell (Table 1). ABBV-176 at a single dose of 0.5 mg/kg was effective (*p* < 0.05) in regressing established tumors, while h16f-MMAE was also efficacious (*p* *<* 0.05) in this model, although at a much higher dose of 3 mg/kg (Fig. 2A). Notably, these and all other in vivo dosing regimens of ABBV-176 in these studies had no significant impact on body weight of the mice compared to controls over the course of the study (unpublished data), nor were there any gross physiologic changes observed to indicate an impact on normal tissues. The non-targeting PBD dimer ADC control (−)Co-PBD exhibited variable activity in the in vivo models, likely driven by high potency of the ADC and tumor related enhanced permeability and retention effects, consistent with previous findings (22 and references therein).

In a patient-derived xenograft (PDX) study with CTG-0869 (0.8x the total PRLR as in the MCF7 cell line), ABBV-176 also exhibited potent anti-tumor activity when compared to vehicle and non-targeted control PBD ADC at the highest dose, 0.5 mg/kg, as well as the 0.3 mg/kg, 0.2 mg/kg, and 0.1 mg/kg doses administered Q7D × 3, which induced durable tumor regression, with similar results seen for CTG-0670 (Fig. 2B and C, (*p* < 0.05), and Table 2). At 0.1 mg/kg, tumors started to regrow by day 63, whereas tumors treated with 0.03 mg/kg had a minimal tumor growth delay. These results are in sharp contrast to the MMAE ADC, which had no impact on this model (Fig. 2B solid blue circles).

**Table 2:** Human Breast Cancer Models ABBV-176; *N* = 3 Screen Study

Model ID	PRLR Density vs MCF7	Drug Doses^**a**^	ABBV-176 Efficacy^**b**^	ER/PR/HER2 BRCA Status
CTG-1124	0.15	0.2	High (84%)	ER+/PR+ BRCA 1 def
CTG-0012	0.75	0.5	High (90%)	TNBC BRCA 1 def
CTG-0869	0.8	0.5	High (93%)	TNBC BRCA 1 mut
CTG-0670	1.64	0.5	High (91%)	TNBC BRCA 1 def, BRCA 2 mut
CTG-0033^c^	2.06	0.2	High (79%)	HER2+ BRCA n.d.
CTG-1171	0.42	0.2	High (82%)	TNBC BRCA 1, 2 mut
CTG-1019	0.37	0.2	High (83%)	TNBC BRCA 1, 2 mut
CTG-1242	3.83	0.2	High (83%)	TNBC BRCA n.d.
CTG-0052	0.46	0.2	Moderate (59%)	TNBC BRCA wild type
CTG-0017	0.27	0.2	Moderate (62%)	TNBC BRCA 1, 2 mut
CTG-1520	0.56	0.5	Low (48%)	TNBC BRCA wild type

*BRCA* breast cancer DNA associated repair gene, *def* deficient, *ER* estrogen receptor, *HER2* human epidermal growth factor receptor 2, *IP* intraperitoneal, *mut* mutation, *n.d.* not determined, *PR* progesterone receptor, *TGI* tumor growth inhibition, *TNBC* triple negative breast cancer

^a^Doses were administered IP Q7D × 3, at the dose level indicated in mg/kg

^b^Low = 25–50% TGI, Moderate = 50–75% TGI, High > 75% TGI compared to control PBD ADC treated group (*p <* 0.0001)

^c^This study was performed with larger size treatment groups (*N* = 6)

**Fig. 3:**
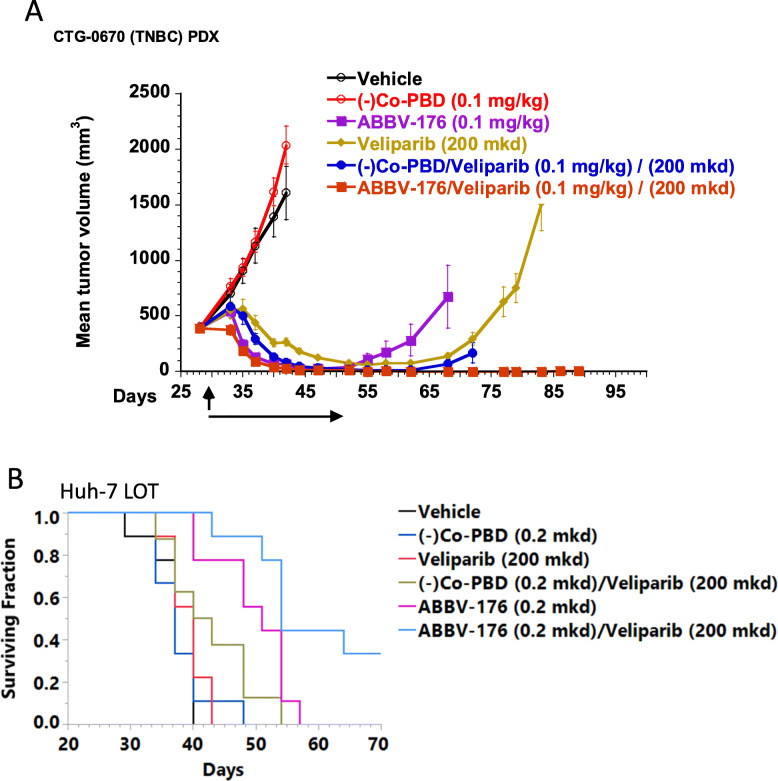
ABBV-176 is active in combination in vivo with the PARP inhibitor Veliparib. Mice with CTG-0670 breast cancer PDX tumors were treated with a single dose of 0.1 mg/kg ABBV-176, 0.1 (−)Co-PBD mg/kg, vehicle, or Veliparib BID × 21 days, or a combination of each ADC with Veliparib at the same amount and schedule (groups of *n* = 10) (**A**). Mice with HuH-7 LOT HCC tumors were treated Q7D × 3 with either 0.2 mg/kg ABBV-176, 0.2 (−)Co-PBD mg/kg, vehicle, or Veliparib BID × 21 days, or a combination of one ADC with Veliparib at the same amount and schedule (groups of *n* = 9). (**B**)

As PDX models have not been grown in cell culture and have not been highly passaged in mice, they are likely to be more closely related to patient tumors. The anti-tumor activity of ABBV-176 was therefore evaluated in an expanded set of primarily, but not exclusively, TNB PDX tumor models that express a range from weak to moderate PRLR. To determine the relative amount of PRLR present in these PDX models, two approaches were used. By Western blot analysis, total PRLR protein levels ranges from 0.15x to 3.83x the total PRLR levels in MCF7, as indicated in the Table 2. Consistent with the total protein levels, when the RNA seq data was compared to TCGA breast cancer data, most of these models expressed low levels of PRLR relative to patient samples, implying that these models express PRLR equal to, or lower than, most breast cancer (BrCa) patient tumors (Fig. 2D-E). Significantly, ABBV-176 was efficacious in 10 of the 12 models tested. To evaluate these responses in more depth, a single dose experiment was performed with CTG-0670. Single dose studies with CTG-0670 demonstrated high potency at 0.1 mg/kg (*p* *<* 0.05), confirming the efficacy of ABBV-176 in BrCa (Fig. 3A). Consistent with the results from the in vitro assay (Table 1), these data indicate that ABBV-176 is a more potent ADC conjugate than auristatin-based ADCs and its activity can extend to lower PRLR-expressing tumors.

To determine the in vivo activity outside of BrCa, the hepatocellular carcinoma line HepG2 was tested for sensitivity to ABBV-176. Despite low PRLR expression (0.09x total PRLR as MCF7), HepG2 had tumor growth delay (*p* < 0.001 compared to vehicle, *p* < 0.005 compared to Co-PBD) when treated with ABBV-176 at 0.2 mg/kg Q7D × 3 as shown in the survival chart (Fig. 2F). Activity was also observed in the hepatocellular carcinoma line Huh-7 LOT (see combination studies described below, and shown in Fig. 3B).

### ABBV-176 combination with PARP inhibition

Maximizing the full clinical potential of ADCs will likely require combinations with other therapeutics. Accordingly, based on complementary mechanisms of action, the effects of combining ABBV-176 with the PARP inhibitor ABT-888 (Veliparib) were investigated in the CTG-0670 TNB BRCA1 deficient, BRCA2 mutant PDX tumor models. In the CTG-0670, one dose at 0.1 mg/kg of ABBV-176 monotherapy treatment was active so the number of CRs was used to compare monotherapy and combination treatment activity shown in Fig. 3A. Combination treatment resulted in increased number of CRs - 8 CRs following combination treatment versus no CRs following monotherapy. Significant tumor growth inhibition was observed through day 89, whereas tumor regrowth was observed following either treatment alone, or treatment with the Veliparib and PBD control combination (Fig. 3A).

To investigate the combinational activity beyond breast cancer models, the activity of ABBV-176 and Veliparib was evaluated in two HCC models, HepG2 and HuH-7 LOT, that express low levels of PRLR (0.09x and 0.26x total PRLR levels in MCF7, respectively). ABBV-176 impacted survival of mice bearing HepG2 tumors, but no significant effect of combination with veliparib was observed (Fig. 2F and data not shown). In contrast, in mice implanted with the higher PRLR expressing HuH-7 tumors LOT, the combination treatment of veliparib and ABBV-176 increased survival (*p* < 0.05) compared to ABBV-176 or (*p* < 0.0001) compared to veliparib alone (Fig. 3B).

## Discussion

New modalities of therapeutic intervention are needed for breast cancer patients, especially for the triple negative subtype, as well as estrogen-independent and relapsed metastatic breast cancers. PRLR is a receptor whose expression is enhanced in breast cancers and presents a potential target for delivering cytotoxic payloads independent of ER, PR or HER2 dependence/expression. Previously described PRLR-targeting therapies include a mutated PRLR ligand, G129L, [32], a neutralizing anti-PRLR antibody, LFA-102, a HER2-PRLR bispecific/ADC approach [5], and a microtubule inhibitor-based ADC, REGN2878-DM1 [4]. In contrast, ABBV-176 is a PRLR-targeting ADC that leverages both the rapid internalization properties of PRLR [5], and a highly potent PBD cytotoxin. Based on results from mouse xenograft tumor models, these properties translate into a highly active therapeutic with activity extending to those tumors with low PRLR expression - as few as 10,000 receptors per cell - which is notably below levels typically present on tumor cells targeted by ADCs. Significantly, ABBV-176 demonstrated anti-tumor efficacy against low PRLR expressing BrCa PDX models. RNA expression levels in these BrCa sensitive PDX tumors corresponded to the lower third of all BrCA tumors within the TCGA database suggesting that the majority of BrCa tumors may express sufficient levels of PRLR to render them sensitive to ABBV-176.

Multiple PRLR positive breast tumor cell lines including triple negative and low PRLR expressers are also sensitive to ABBV-176. The efficacy of ABBV-176 was superior compared to the h16F- MMAE ADC with potent activity observed in PDX models including some that were completely refractory to h16-MMAE at ten-fold higher doses. In vitro and in vivo anti-tumor activity was also seen with ABBV-176 in hepatocellular carcinoma cell lines refractory to the MMAE ADC. Although PRLR expression has generally been primarily associated with breast cancers, additional evaluation of the efficacy of ABBV-176 in non-breast cancer indications that express low levels of PRLR may be warranted.

A key challenge with therapeutic ADCs is the potential for on-target and off-target toxicities. In the case of ABBV-176, these concerns are exacerbated by the potent PBD payload that necessitates lower therapeutic doses in patients and subsequently reduced exposures as shown in [33]. This in turn may limit ADC availability to the tumor site especially if normal tissue disposition is observed. Ultimately combination approaches may be needed to minimize the dose levels needed of any single agent to minimize each individual therapy’s toxicities, while maximizing clinical benefit.

The PBD cytotoxin cross links DNA and causes DNA breaks, requiring DNA repair mechanisms for cell survival. Poly ADP ribose polymerase (PARP1) binds to DNA breaks and is critical for base excision repair and nucleotide excision repair pathways, and PARP inhibitors are approved for treatment of BrCa patients with germline *BRCA* mutations [34]. This mechanism of action suggests that PARP inhibitors such as Veliparib may enhance the activity of PBD-dimer ADCs. The combination of ABBV-176 and the PARP inhibitor Veliparib demonstrated enhanced activity in the *BRCA1* deficient triple negative breast model CTG-0670 as well as the liver CDX HuH-7 LOT model, but not the HepG2 liver model. Due to a mutation in the FANCC gene, HuH-7 cells have impaired FANCD2 nuclear foci-formation in response to irradiation, while HepG2 cells do not. After DNA damage, FANCD2 and FANCI are activated by monoubiquitination to complex with other Franconi anemia proteins, BRCA2, RAD51, and aditional DNA damage repair proteins, critical for repair of interstrand crosslinks. The damage caused by the PBD delivered by ABBV-176, coupled with this DNA repair impairment, could drive the PARP inhibition combination effects in the absence of BRCA-deletions [35, 36]. Recently, Zhong et al. has also reported improved antitumor effects of a 5 T4-PBD ADC when combined with the PARP inhibitor Oliparib [37]. ABBV-176 therefore offers an expanded breadth of efficacy, potentially even beyond breast cancer, while combination with potentiators of DNA damage offers opportunities to reduce dose levels and toxicities and potentially enhance efficacy in patients.

## Conclusions

ABBV-176 is a potential therapeutic for metastatic breast cancer patients that have lost sensitivity to ER-targeting modalities and as well those that relapse after HER2-based approaches such as Herceptin, Kadcyla patients. ABBV-176 binds to PRLR, is rapidly internalized, and delivers a potent PBD cytotoxin to tumor cells. This DNA crosslinking payload offers a distinct mechanism of action from BrCa standard of cares that include anti-estrogens, microtubule inhibitors, CDK4/6 inhibitors, and HER2 targeting therapies, that combines with PARP inhibition and can contribute to circumventing resistance to these treatments.

## Supplementary Information

**Additional file 1: Fig. S1.** Western blot analysis for PRLR in normal/nontransformed human cells. Panel A: HUVEC (umbilical vein endothelium), PrEC (prostate), HUF (primary uterine fibroblasts), NHBE (bronchial epithelium), HMEC (mammary epithelium), HRE (primary renal mixed epithelium), HRMC (renal mesangial cells), BT474 (breast cancer) as a positive control. Panel B: MCF10A (normal breast line), HMVEC (microvascular endothelium), and THLE-3 (immortalized liver). Examples of In vitro cell killing assay. Panel C: HMEC cells. Panel D: HRMC cells.

**Additional file 2: Fig. S2.** Full Western blots from S1. Panels A and B: M are markers, X are irrelevant cell lines, and remaining lanes as in S1.

## Data Availability

All data generated or analysed during this study are included in this published article and its supplementary information files.

## Abbreviations

PRLR: Prolactin receptor
ADC: Antibody-drug conjugate
MMAE: Monomethyl auristatin
PBD: Pyrrolobenzodiazepine
PARP: Poly-ADP ribose polymerase
TNBC: Triple negative breast cancer
HER2: Human epidermal growth factor receptor 2
PRL: Prolactin
ECD: Extracellular domain
FACS: Fluorescence activated cell sorting
ELISA: Enzyme enzyme-linked immunosorbent assay
(−)Co: Non targeted human IgG
PDX: Patient-derived xenograft
mkd: Milligrams per kilogram per day
Q7D: Once every 7 days dosing
BrCa: Breast cancer
HCC: Hepatocellular carcinoma
IP: Intraperitoneal
mut: Mutation
n.d.: Not determined
ER: Estrogen receptor
PR: Progesterone receptor
TGI: Tumor growth inhibition
BID: Twice daily
