# Supplementary material for: ABBV-176, a PRLR antibody drug conjugate with a potent DNA-damaging PBD cytotoxin and enhanced activity with PARP inhibition
Source: BMC Cancer. 2021 Jun 9;21:681. doi: 10.1186/s12885-021-08403-5 (PMC8191021; doi:10.1186/s12885-021-08403-5)
Supplement: Supplementary file 1 — Additional file 1: Fig. S1. Western blot analysis for PRLR in normal/nontransformed human cells. Panel A: HUVEC (umbilical vein endothelium), PrEC (prostate), HUF (primary uterine fibroblasts), NHBE (bronchial epithelium), HMEC (mammary epithelium), HRE (primary renal mixed epithelium), HRMC (renal mesangial cells), BT474 (breast cancer) as a positive control. Panel B: MCF10A (normal breast line), HMVEC (microvascular endothelium), and THLE-3 (immortalized liver). Examples of In vitro cell killing assay. Panel C: HMEC cells. Panel D: HRMC cells. [file 12885_2021_8403_MOESM1_ESM.pptx]

## Slide 1
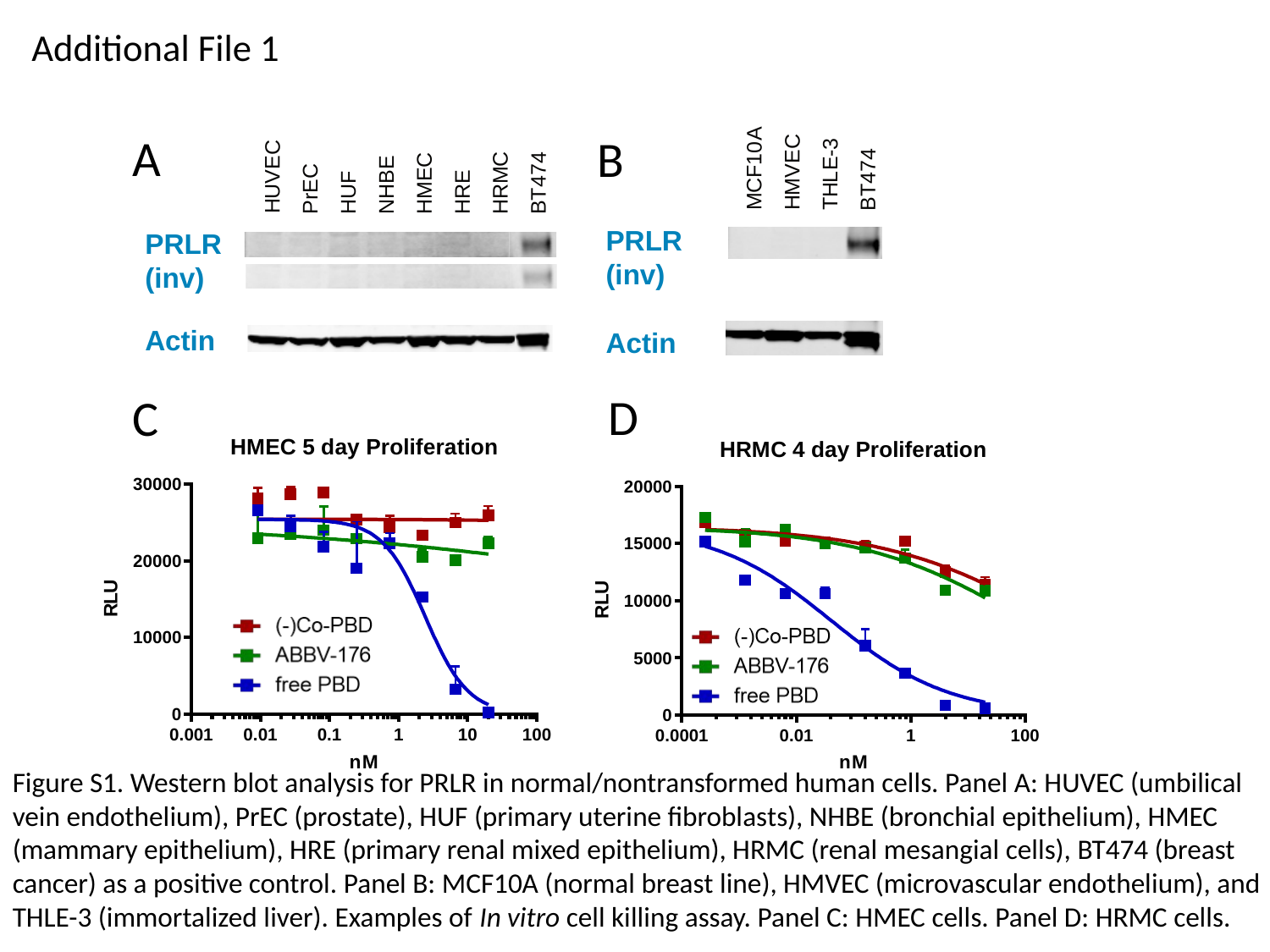

HUVEC
PrEC
HUF
NHBE
HMEC
HRE
HRMC
BT474
# Additional File 1
MCF10A
HMVEC
THLE-3
BT474
A
B
PRLR
(inv)
PRLR
(inv)
Actin
Actin
D
C
Figure S1. Western blot analysis for PRLR in normal/nontransformed human cells. Panel A: HUVEC (umbilical vein endothelium), PrEC (prostate), HUF (primary uterine fibroblasts), NHBE (bronchial epithelium), HMEC (mammary epithelium), HRE (primary renal mixed epithelium), HRMC (renal mesangial cells), BT474 (breast cancer) as a positive control. Panel B: MCF10A (normal breast line), HMVEC (microvascular endothelium), and THLE-3 (immortalized liver). Examples of In vitro cell killing assay. Panel C: HMEC cells. Panel D: HRMC cells.
