# Supplementary material for: ABBV-176, a PRLR antibody drug conjugate with a potent DNA-damaging PBD cytotoxin and enhanced activity with PARP inhibition
Source: BMC Cancer. 2021 Jun 9;21:681. doi: 10.1186/s12885-021-08403-5 (PMC8191021; doi:10.1186/s12885-021-08403-5)
Supplement: Supplementary file 2 — Additional file 2: Fig. S2. Full Western blots from S1. Panels A and B: M are markers, X are irrelevant cell lines, and remaining lanes as in S1. [file 12885_2021_8403_MOESM2_ESM.pptx]

## Slide 1
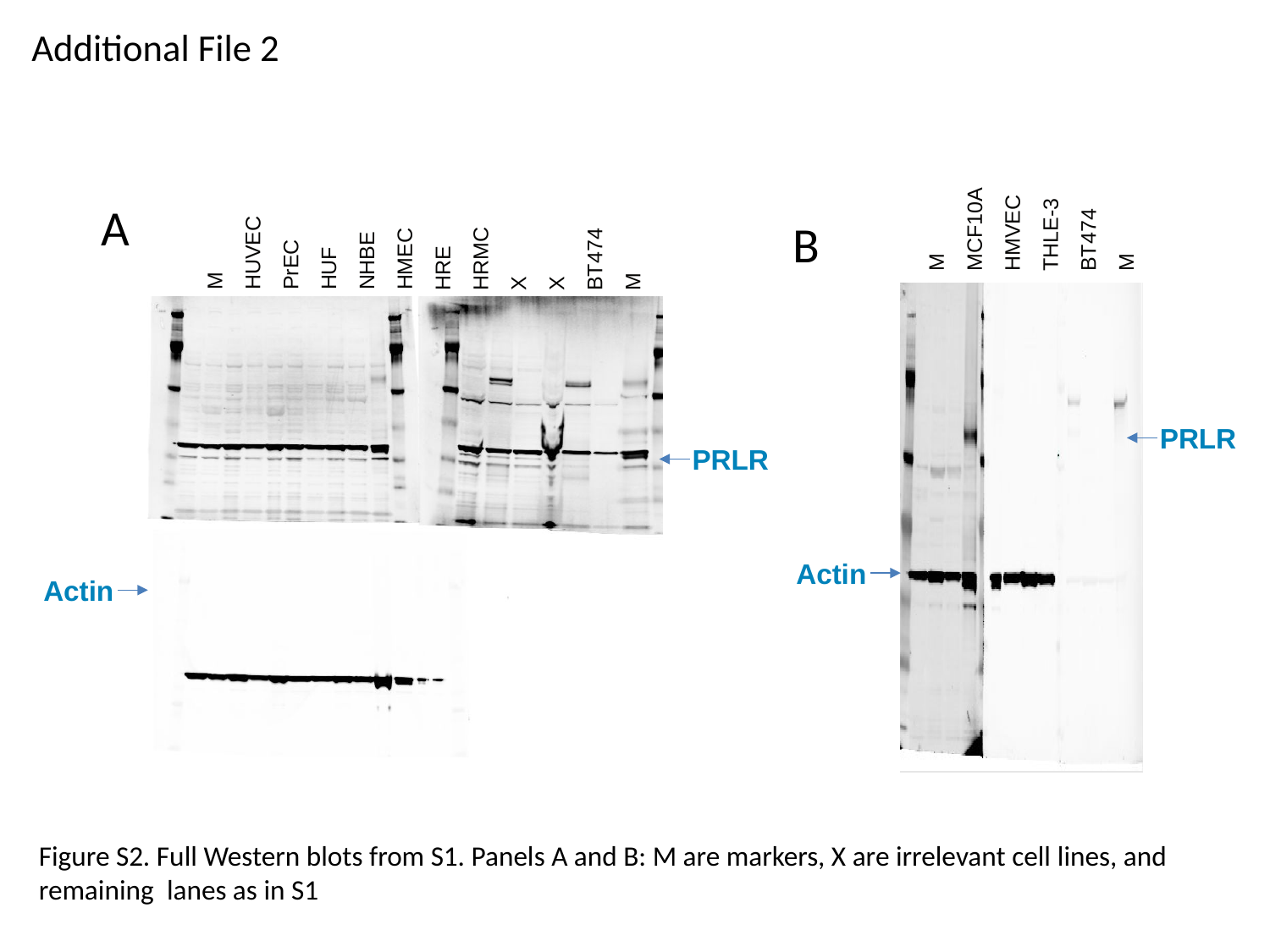

M
HUVEC
PrEC
HUF
NHBE
HMEC
HRE
HRMC
X
X
BT474
M
# Additional File 2
M
MCF10A
HMVEC
THLE-3
BT474
M
A
B
PRLR
PRLR
Actin
Actin
Figure S2. Full Western blots from S1. Panels A and B: M are markers, X are irrelevant cell lines, and remaining lanes as in S1
